# Mixed Micellization, Thermodynamic and Adsorption Behavior of Tetracaine Hydrochloride in the Presence of Cationic Gemini/Conventional Surfactants

**DOI:** 10.3390/gels8020128

**Published:** 2022-02-17

**Authors:** Naved Azum, Malik Abdul Rub, Anish Khan, Maha M. Alotaibi, Abdullah M. Asiri, Mohammed M. Rahman

**Affiliations:** 1 Center of Excellence for Advanced Materials Research, King Abdulaziz University, Jeddah 21589, Saudi Arabia; navedazum@gmail.com (N.A.); malikrub@gmail.com (M.A.R.); anishkhan97@gmail.com (A.K.); aasiri2@gmail.com (A.M.A.); 2 Chemistry Department, Faculty of Science, King Abdulaziz University, Jeddah 21589, Saudi Arabia; mmsalotaibi@kau.edu.sa

**Keywords:** anesthetic drug, novel surfactant, mixed micelle, synergism

## Abstract

In this approach, tensiometry and UV-visible techniques are used to determine the effect of cationic gemini and conventional surfactants on tetracaine hydrochloride (TCH), an anesthetic drug. We have estimated micellar, interfacial, and energetic constraints. To gain a deep understanding of their mixed association behavior, the outputs were examined using different theoretical models. The critical micelle concentration for single and mixed amphiphiles was estimated. The cmc values of mixed amphiphiles were found between the individual amphiphiles due to strong attractive interaction (synergism) between the components after mixing. The non-ideal behavior of mixtures was confirmed by the larger values of ideal cmc than the experimental cmc values. The negative values of interaction parameter (*β*) and values of activity coefficients less than unity indicate strong synergistic interaction between drug and surfactant. The stability of the mixed systems is demonstrated by the negative Gibbs free energy of micellization and excess free energy of micellization. In contrast to a single chain surfactant, a double chain surfactant (gemini) exhibits better interactions with the drug. Spectral measurements (UV-visible spectra) were used to monitor the binding of the drug with surfactant (conventional as well as gemini). Studying these mixed aggregates could help to optimize their compositions and find synergistic properties between TCH monomers and surfactants.

## 1. Introduction

**Figure 1:**
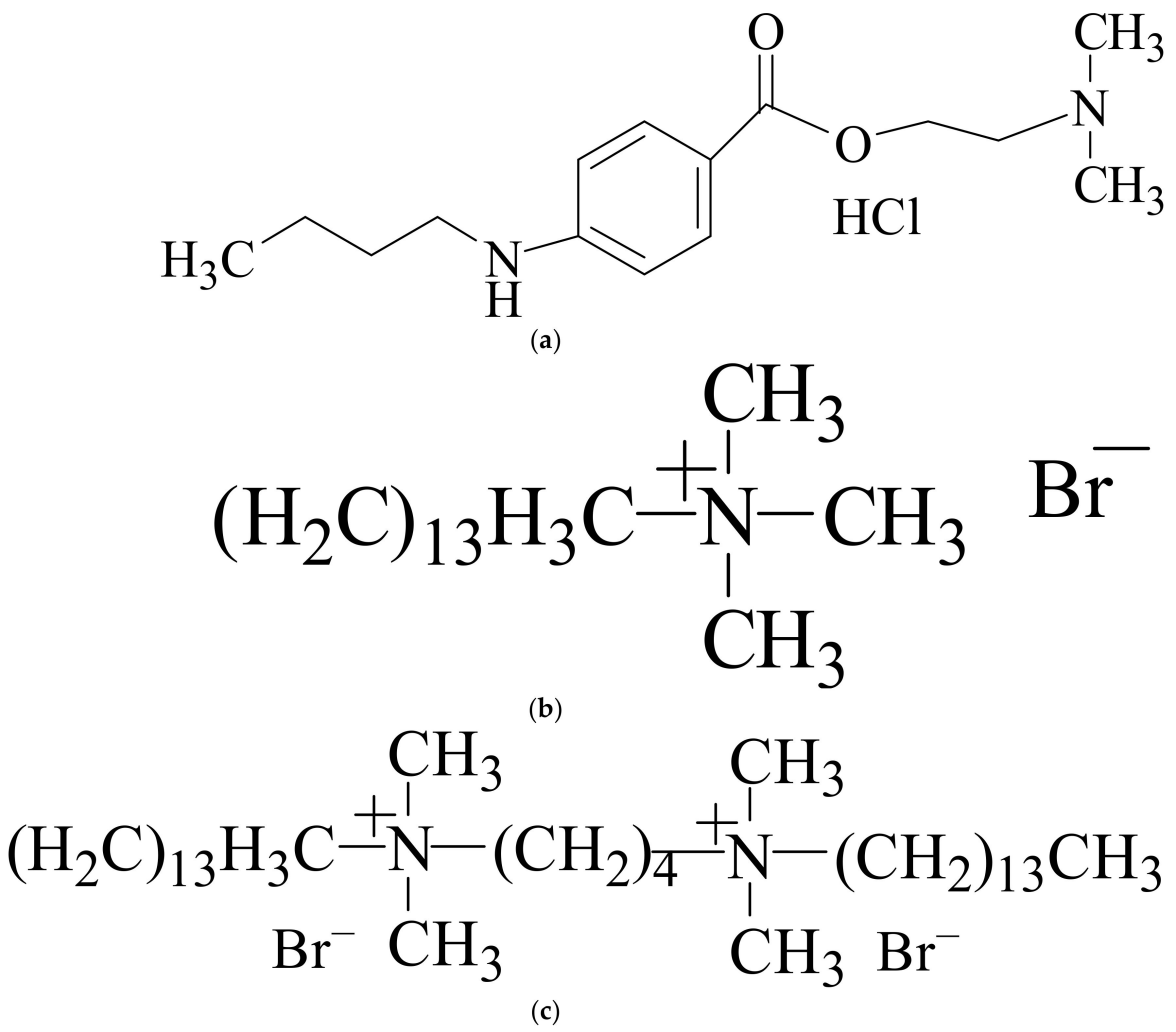
Examined amphiphiles (**a**) TCH, (**b**) TTAB, and (**c**) 2G4.

The investigation of drug-surfactant or drug-polymer interactions can tell us about drug-protein interactions and the solubilizing properties of micelles. Thus, the drug-surfactant interaction is very important from a pharmaceutical and pharmacological standpoint, that is why their use has increased in recent years [1,2,3,4,5,6,7,8,9,10]. Tetracaine hydrochloride (TCH) is a benzoate ester compound made up of 4-N-butyl benzoic acid and 2-(dimethylamino) ethanol (Figure 1) act as a local anesthetic drug for surface and spinal anesthesia. TCH is also available in combination with lidocaine that is used as a local anesthetic cream. In surgery or other similar procedure, TCH blocks the pain signals by nerve endings [11,12]. The interaction between the anesthetic molecule and lipid molecules of the biomembrane at the interface is responsible for the anesthetic action of the drug. Hence, different physicochemical properties of mixtures are supposed to play a vital role in this mechanism.

Among all the drug administration routes, oral one is the economic, appropriate, and regularly utilized drug administration mode [13]. Though, hydrophobicity (less water-soluble) of drug molecules reduces the efficiency of the oral route and creates some undesirable side effects in the human body [14]. The drug permeability across the cell membrane is also diminished by the low solubility. About 70% of compounds discovered for pharmaceutical applications are poor water soluble [14]. To overcome this problem an appropriate transporter or carrier is used that enhances drugs solubility and permeability. Different types of compounds, i.e., surfactants, block co-polymers, cyclodextrin, etc. are being used as a carrier [15,16,17,18,19]. However, among these, the surfactants aggregates are the most favorable aspirants because of extraordinary physicochemical properties, formation of nano-size micelles, easy preparation, high permeability, high solubility, and stability. The extraordinary action of surfactant is due to its ability to solubilize the poorly water-soluble drug in micellar core and transportation of drug to the precise locations. Therefore, surfactants can lessen the harmful side effects of the drug and enhance its bioavailability.

Surface active agents, or surfactants, are a special kind of material that can reduce interfacial tension, form micelles, colloid systems in solutions, that create a different microenvironment in a bulk solution when dissolved in water or some other solvents. Surfactants from an earlier age are being widely utilized in domestic as well as industrial purposes [19,20,21,22,23]. Nowadays surfactants influence the majority of phases of our daily life, either directly in household detergents and personal care products or indirectly in the production and processing of the materials which surround us. At very high concentrations surfactants can form gel-type structures due to dense packing and hexagonal, cubic, or lamellar phases are formed. For the last few years, gemini surfactants are in limelight because of their superior properties to conventional surfactants. Gemini surfactants make micelle at a much low concentration than a conventional one. In addition, they have better interfacial activity, higher hydrophobicity, better wetting properties [24,25,26]. Among the gemini surfactants, cationic ones are antifungal, antibacterial, and antiseptic, which have attracted attention concerning their interaction with DNA and lipids. Recently alkyl polyoxyethylene sulfonate gemini surfactants have been synthesized by Yan et al. [27] that have excellent emulsification ability. The self-assembly of these surfactants can lead to a variety of colloidal structures.

Here in this paper, we used an amphiphilic drug, TCH as a model drug, and conventional and gemini surfactants as drug excipients. A large number of drug-like TCH used in pharmaceutical formulations are amphiphilic [15,28,29,30,31]. Due to the presence of dual characters such as a surfactant molecule they are also associated with small, organized aggregates known as a micelle. The TCH and gemini mixtures not only show synergism but also give antibacterial activity against gram-positive bacteria [32]. Thus, these mixtures may offer advancements in biomedical and pharmaceutical fields. Micelles provide several functional characteristics of the amphiphilic drug in the attendance of other pharmaceutical excipients. The micelles provide regions of different physicochemical properties. The low polarity environment is created by the hydrophobic core while high charge concentration (ionic surfactants) or high polarity (nonionic surfactants) regions are created by the head groups. When an amphiphilic drug is incorporated into the micelle of surfactant via self-association or by other means [23], drug physicochemical properties (reaction rates, reaction mechanism, degree of ionization, etc.) may be affected.

**Figure 2:**
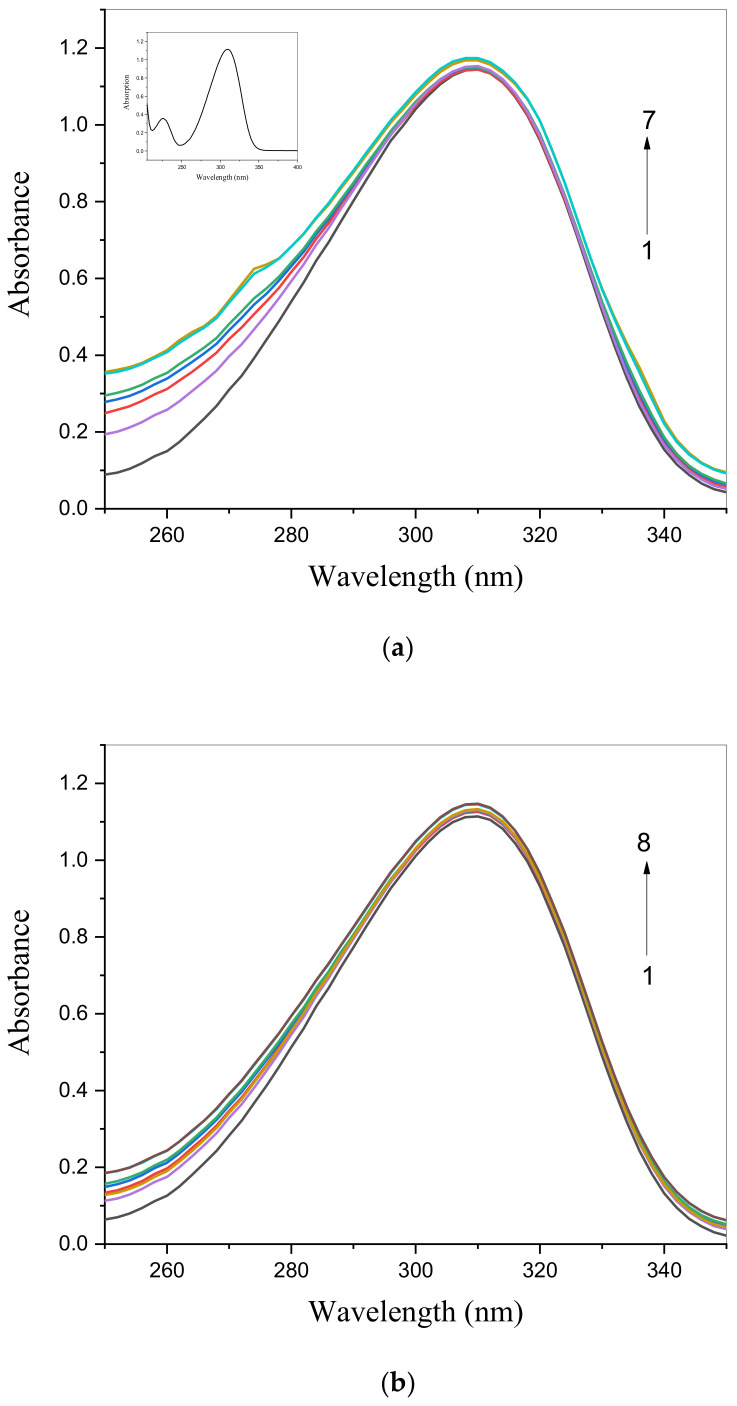
The UV-visible spectrum of TCH with the increasing concentration of (**a**) TTAB, and (**b**) 2G4. The inset in (**a**) shows the UV-visible spectra of TCH alone.

The aim of this work is the future use of such novel micelles as a drug carrier that will be less toxic and cost-effective. Before doing the further advanced study, the physicochemical characterization of drug-surfactant mixtures is needed. We studied here two types of combination, (i) drug + conventional surfactant, (ii) drug + gemini surfactant. The conventional surfactant (tetradecyl trimethyl ammonium bromide, TTAB) a monomeric part of gemini (*N*, *N*′-bis(dimethyl tetradecyl)-α,ω-alkane diammonium bromide, 2G4) is used (Figure 1). In literature, some studies are available on drug-surfactant interaction via different techniques but very few studies are with gemini surfactants. The various interfacial and bulk parameters were computed by tensiometeric titration. Various theoretical models (Clint, Rubingh, Rosen, Motomura) assisted to compute these parameters. Finally, UV-visible spectroscopy was employed to identify potential relations among the drug and surfactant mixture (Figure 2).

## 2. Results and Discussion

### 2.1. Mixed Micellization at Bulk Phase

#### 2.1.1. Aggregation of Amphiphiles

**Figure 3:**
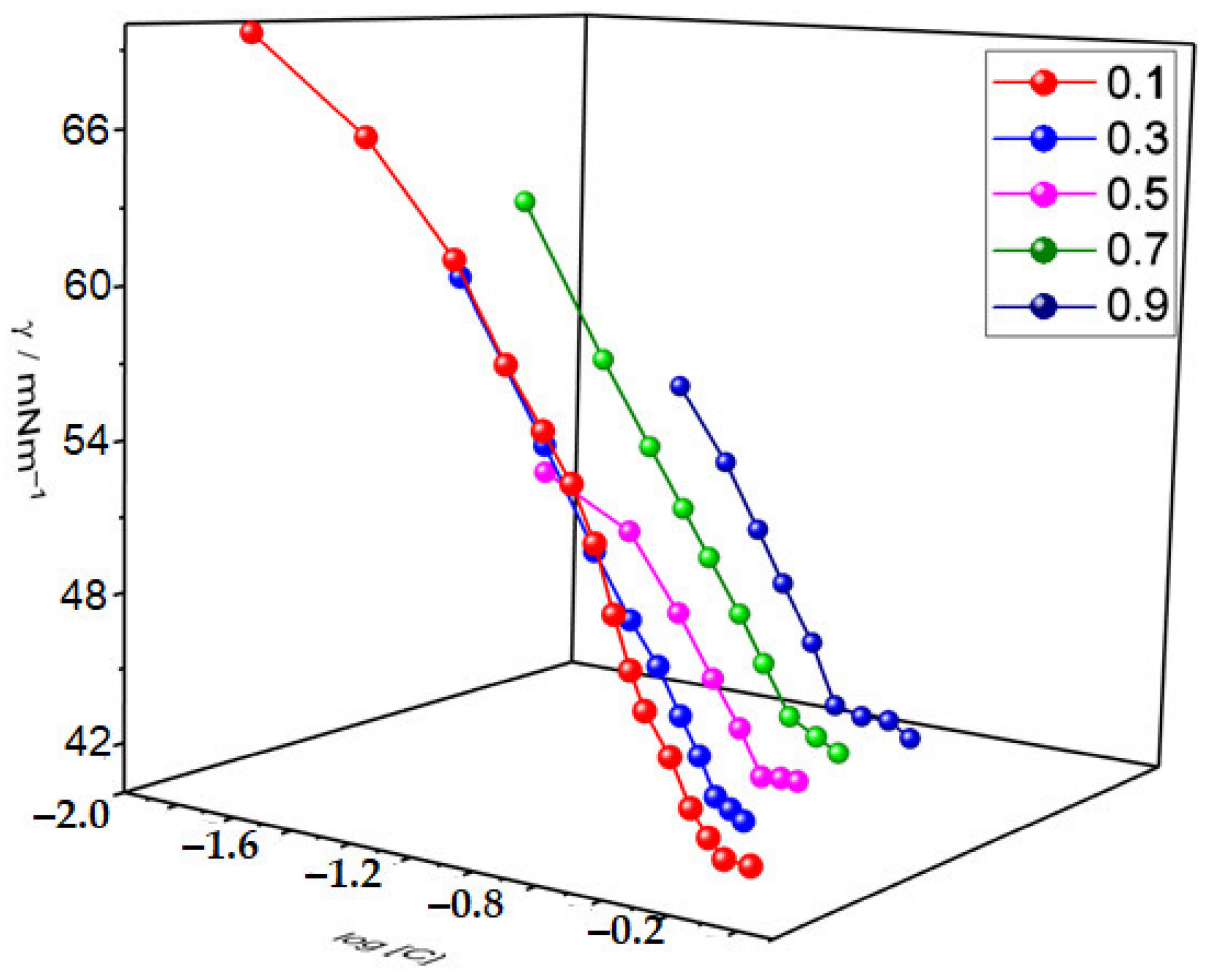
Surface tension (γ) vs. log molar concentration (C) of 2G4 + TCH mixed systems (gemini surfactant (2G4); tetracaine hydrochloride (TCH)).

**Scheme 1:**
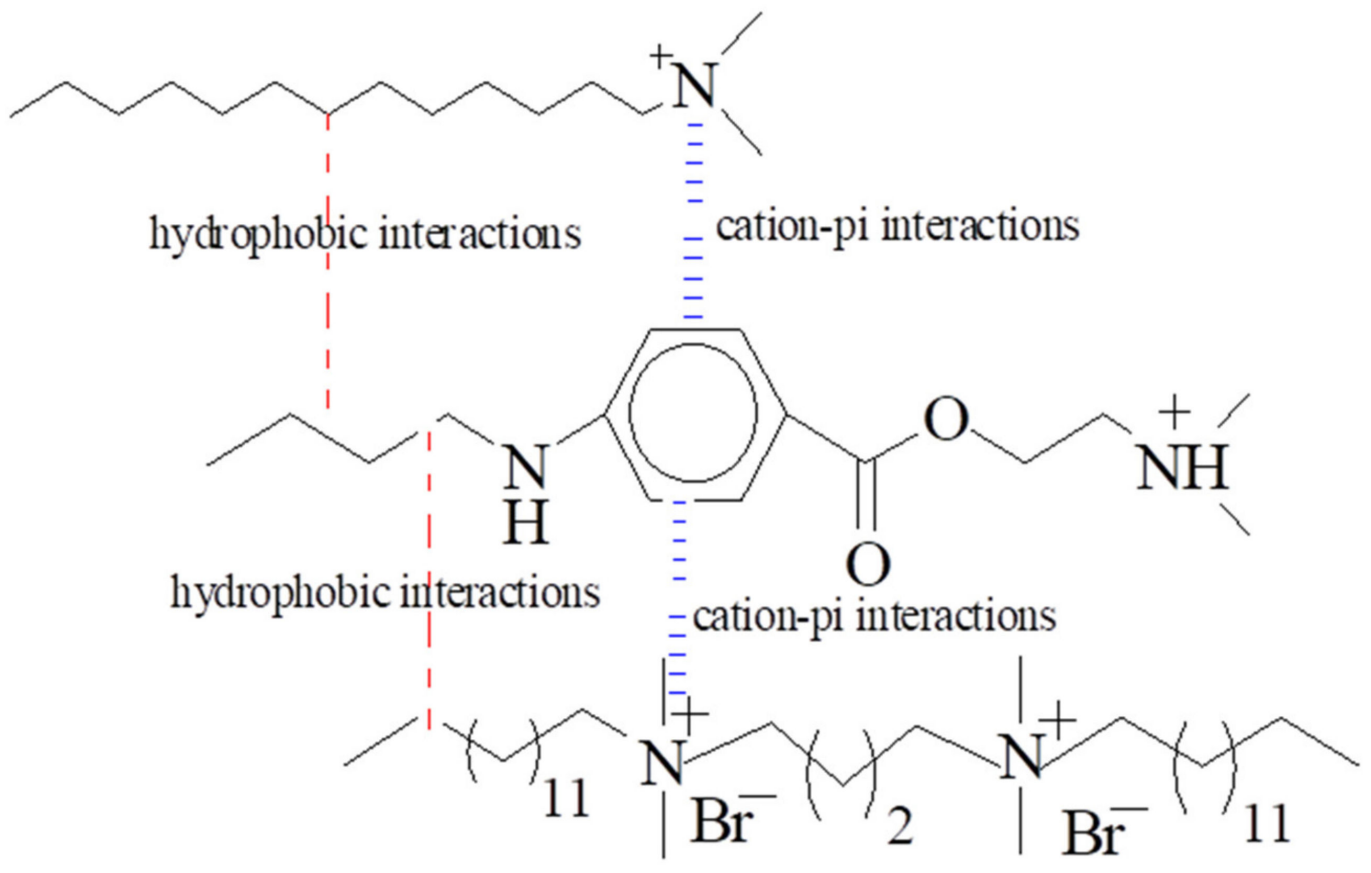
Schematic illustration of interactions taking place among tetracaine hydrochloride and conventional/gemini surfactants.

When surfactants are present in low concentrations then most of the monomers roam freely in the solution and behave similar to simple electrolytes. At above a particular concentration, called critical micelle concentration (cmc) they try to aggregate and form a globular structure (micelle) [33,34]. The surface tension of single and drug-surfactants mixtures has been computed by tensiometric titration. Figure 3 represents the variation of surface tension vs. molar concentration of mixed amphiphiles at 298.15 K. There are mainly two factors that are responsible for micelle formation (i) electrostatic interactions operating between the charged head group and (ii) hydrophobic interaction among tails of interacting components. Herein, the drug and surfactants have the same charge on head groups so the second factor is operating along with cation–pi interactions (Scheme 1). The lower cmc values of mixtures than individual amphiphiles signify favorable mixing (Table 1). The surfactant chain length is a major driving factor for micellization, and hydrophobic interaction is the major driving force. During the micelle formation water molecules in the hydration shell around the chains length of surfactant molecules are released and entropy increases. As more water molecules are released if the chain lengths increase, resulting in more entropy increases, hence micellization occurs at a lower concentration. The values of cmc are listed in Table 1. The cmc values of pure amphiphiles are well-matched with earlier studies [19,30,35]. The lower cmc value of gemini surfactant among these amphiphiles confirms the better propensity of gemini surfactant for the formation of micelle.

#### 2.1.2. Interaction of Drug with Surfactants in the Bulk

By applying the pseudo phase separation model, the non-ideality between two mixed amphiphiles is treated by considering the chemical potential of individual amphiphiles. For two real amphiphiles mixed systems, the critical micelle concentration can be computed with help of *cmc_s_* (*cmc*_1_ and *cmc*_2_) and mole fractions ($\alpha_{1}$ and $\alpha_{2}$) of individual amphiphiles as:

(1) $$\frac{1}{cmc^{*}}=\frac{\alpha_{1}}{f_{1}cmc_{1}}+\frac{\alpha_{2}}{f_{2}cmc_{2}}$$

On the other hand, for an ideal mixed amphiphilic system, the *f*_1_ = *f*_2_ = 1. To check the ideality of mixed system, the ideal critical micelle concentration for the mixed system was computed by using Clint equation based on pseudo phase thermodynamic model [36]:

(2) $$\frac{1}{cmc^{*}}=\frac{\alpha_{1}}{cmc_{1}}+\frac{\alpha_{2}}{cmc_{2}}$$

The above Clint equation is helpful to understand the ideal behavior of mixed amphiphilic systems. Table 1 shows that the experimental cmc values diverge negatively with ideal values. The non-ideal behavior of the TCH + TTAB/2G4 mixture may be due to the favorable interactions between the hydrophobic chains of amphiphiles that make the mixed system more favorable than expected for the ideal one.

However, for the non-ideal mixed system, a new model is developed named as Rubingh model [37]. Rubingh model based on regular solution approximation (RST) relates the activity coefficients of components with micellar mole fraction of component 1 ($X_{1}^{Rub}$) as:

(3) $$f_{1}^{Rub}=exp[\beta^{Rub}{\left(1-X_{1}^{Rub}\right)}^{2}]$$

(4) $$f_{2}^{Rub}=exp[\beta^{Rub}{\left(X_{1}^{Rub}\right)}^{2}]$$

The values of these $f_{1}^{Rub}$ and $f_{2}^{Rub}$ are given in Table 1. The values of both these two parameters are found to be lower than 1 indicating the presence of non-ideality among the mixing components. With the help of Equations (1)–(4) the values of $X_{1}^{Rub}$ easily deduced by the following equation:

(5) $$\frac{{\left(X_{1}^{Rub}\right)}^{2}\ln\left(\alpha_{1}cmc/X_{1}^{Rub}cmc_{1}\right)}{{\left(1-X_{1}^{Rub}\right)}^{2}\ln\left[\left(1-\alpha_{1}\right)cmc/\left(1-X_{1}^{Rub}\right)cmc_{2}\right]}=1$$

For an ideal mixed system the micellar mole fraction of component 1 can be expressed as [38]:

(6) $$X_{1}^{\mathrm{ideal}}=\frac{\alpha_{1}cmc_{2}}{\alpha_{1}cmc_{2}+\alpha_{2}cmc_{1}}$$

The values of $X_{1}^{Rub}$ obtained from Equation (5) by iterative method and $X_{1}^{\mathrm{ideal}}$ are listed in Table 1. For current mixed systems the values $X_{1}^{Rub}$ are lower than $X_{1}^{\mathrm{ideal}}$ indicates that the mixed systems contain fewer surfactants molecules than in the ideal mixed state, with more transfer of TCH molecules from solution to micellar phase. The difference in the values of these two parameters was slightly more in the case of the TCH + 2G4 mixed system due to the greater hydrophobicity of gemini surfactant than its counterpart (TTAB).

In Equations (3) and (4) a term $\beta^{Rub}$ was used that determine the interaction between two amphiphiles in a mixed system. This interaction parameter $\beta^{Rub}$ can be computed by using Equation (7):

(7) $$\beta^{Rub}=\frac{\ln\left(\alpha_{1}cmc/X_{1}^{Rub}cmc_{1}\right)}{{\left(1-X_{1}^{Rub}\right)}^{2}}$$

In Equation (7) the $X_{1}^{Rub}$ stands for micellar mole fraction of components 1. Table 1 contained the values of $\beta^{Rub}$. In a mixed surfactants system, the nature and strength of interaction can be judged by the values of $\beta^{Rub}$. The positive values of $\beta^{Rub}$ are associated with the positive deviation from ideality or antagonistic behavior between surfactants monomers. Negative values $\beta^{Rub}$ show the non-ideal behavior or synergistic behavior between two surfactants. If the value of $\beta^{Rub}$ is zero, which means there is no interaction. The reduction in free energy of micellization is associated with the higher absolute value of $\beta^{Rub}$, that makes the process thermodynamically more stable. For the current TCH + TTAB/2G4 mixed system the $\beta^{Rub}$ values are negative, confirming the synergistic behavior between drug and surfactants molecules. The negative values of $\beta^{Rub}$ remain consistent throughout the mole fractions for both mixed systems and its magnitude indicates the degree of non-ideality. The negative values of $\beta^{Rub}$ increases with the mole fraction of component 1 in the mixed system because of increasing the hydrophobic interaction among the tail parts of surfactants and drug. Expect hydrophobic interaction the π electrons of the benzene ring of drug and cation of surfactants are also responsible for increasing values of $\beta^{Rub}$ (Scheme 1). Table 1 also reveals that the interactions present in the TCH + 2G4 mixture are more than TCH + TTAB due to the lower cmc values of 2G4 and higher hydrophobicity as compared to TTAB. Ali et al. [39] have also been reported similar behavior.

#### 2.1.3. Thermodynamic Parameters in the Bulk Phase

The free energy change for the binary mixed system can be computed by using regular solution theory (RST) as follows [20,40,41,42]:

(8) $$\Delta G_{m}=RT[X_{1}^{Rub}\ln\left(X_{1}^{Rub}f_{1}^{Rub}\right)+X_{2}^{Rub}\ln\left(X_{2}^{Rub}f_{2}^{Rub}\right)]$$

For an ideal binary mixed system, the values of activity coefficients ($f_{1}^{Rub}$ and $f_{2}^{Rub}$) are equivalent to 1, then Equation (8) for free energy change for the ideal binary mixed system can be given by:

(9) $$\Delta G_{m}^{\mathrm{ideal}}=RT[X_{1}^{Rub}\ln X_{1}^{Rub}+X_{2}^{Rub}\ln X_{2}^{Rub}]$$

For our binary mixed systems, the free energy change values are given in Table 2. It is confirmed from data that the values are negative, which indicates the spontaneity of mixed micelle formation and micelle formed are stable. The values of $\Delta G_{m}$ are showing negative deviation from $\Delta G_{m}^{\mathrm{ideal}}$, favor the formation of real mixed micelle formation instead of an ideal one. In the literature, the same behavior was confirmed by the investigators [43,44].

Regular solution theory is also used to compute the excess free energy of mixed systems ($G_{mix}^{E}$) or enthalpy change ($\Delta H_{m}$) for the binary mixed system by the following relation [41,44]:

(10) $$G_{mix}^{E}=\Delta H_{m}=RT[X_{1}^{Rub}\ln f_{1}^{Rub}+X_{2}^{Rub}\ln f_{2}^{Rub}]$$

With the help of Equations (8) and (10), the entropy change for the mixed system is also computed as:

(11) $$\Delta S_{m}=\frac{\Delta H_{m}-\Delta G_{m}}{T}$$

For current binary mixed systems, the positive values of entropy confirmed the entropy contribution must be the driving force of mixed micellization (Table 2). The same results were also given in the literature [45,46]. For the 2G4 + TCH mixed system, the entropy contribution is more at all more fractions. This may be due to the more attractive interaction (synergistic interaction) between two amphiphiles at mixed state, which may facilitate the formation of well-organized mixed micelle. The absolute values of entropy/free energy change are more than 0.5, which means that the mixed micelle formation is an entropically favorable process.

### 2.2. Mixed Micellization at Interface or Surface

#### 2.2.1. Interfacial Properties at the Air-Water Interface

As earlier in the above section we have mentioned that the cmc values of single and mixed amphiphilic systems are calculated by the intersection of two lines before and after cmc (Figure 3). From these observations, we were able to calculate various surface parameters for single and mixed amphiphiles. The Gibbs adsorption equation was used to calculate the surface excess at the interface by the following relation [47]:

(12) $$\Gamma_{max}=-\frac{1}{2.303nRT}\left(\frac{d\gamma }{dlogC}\right)$$

For TTAB and TCH the values of *n* are taken as 2 and for gemini surfactant we put the value of *n* = 3. For the current mixed system, the values of *n* were calculated by using $X_{1}^{s}$*n*_1_ + $X_{2}^{s}$*n*_2_ expression [47,48]. Where $X_{1}^{s}$ and $X_{2}^{s}$ are the mole fraction of components 1 and 2 at interface. The values of surface excess determine the amount of surface-active compound adsorbed at per unit area of surface or interface. Higher the values of $\Gamma_{max}$ means more crowding and stuffing of amphiphilic molecules at the surface. The squeezing or stuffiness of the molecules is beneficial for some interface qualities such as foaming, emulsification, and wetting. The values of $\Gamma_{max}$ were used to calculate the minimum area per molecule [49]:

(13) $$A_{min}=\frac{10^{20}}{N_{A}\Gamma_{max}}\;({Å}^{2})$$

where Avogadro’s number is designated by the symbol, *N*_A_. The values of $\Gamma_{max}$ and $A_{min}$ are listed in Table 3. The knowledge of the degree of packing and the orientation of amphiphiles adsorbed at the interface is governed by the area per molecule at the surface. The $A_{min}$ values depend on the chain length of amphiphiles. Gemini surfactant has a higher chain length among the other two amphiphiles (higher $A_{min}$ value), so the gemini surfactant has a higher packing density at the air-water interface. The same behavior of gemini surfactant is also found in the literature [47,50]. For the TTAB + TCH mixed system the values of $A_{min}$ of mixtures are lower than the drug, this show that more tightly packed amphiphiles curvature is formed. For 2G4 + TCH mixed system, the values of $A_{min}$ of mixtures are higher than the TCH, indicating a loosely packed monomer interface is formed. When the chain lengths of amphiphiles are different, the one component above the height of the adjacent component will create thermal motion. Due to this thermal motion, the interface is disturbed and as a result, loose packing is created ($\Gamma_{max}$ decreases and $A_{min}$ increases). The increase or decrease of these two values depends on various factors for instance host head group charges, modes of bonding between components, presence of components at the surface. The decline of surface activity of TCH in the presence of 2G4 maybe because both are positive charge and the bulkiness of 2G4 remains these two molecules separate from each other at the interface and the space between these two amphiphile increases ($A_{min}$ increases). The values of $A_{min}$ of drug with TTAB decrease because hydrophobic interactions among structurally similar head groups (positive-positive head group). This factor suppresses the effect of similar head groups and decreases the $A_{min}$. Sheikh et al. [51] also observed the same explanation.

When the surface tension value of pure water is reduced by about 20 dyn/cm by the adsorption of amphiphiles, the surface concentration is 84–99.9% saturated. Thus, the efficiency of adsorption of an amphiphile is measured by the depression of surface tension of water by 20 m Nm^−1^ on the addition of an amphiphile. Therefore, it is the least concentration required to create saturation adsorption at the surface [52]. Adsorption efficiency ($pC_{20}$) calculated by the equation:

(14) $$pC_{20}=-logC_{20}$$

Table 3 listed the values of $pC_{20}$ that are related to the efficiency of adsorption of surfactants at the interface. The present drug and surfactants mixtures have lower values of $pC_{20}$, that shows the mixtures are at a lower efficiency than the drug.

Rosen and Hua [48] gave a thermodynamic model that relates the molar concentration of two amphiphiles in the solution phase with mole fraction of components ($X_{1}^{s}$ & $X_{2}^{s}$) at interface as:

(15) $$C_{1}=C_{1}^{0}f_{1}^{S}X_{1}^{S}$$

(16) $$C_{2}=C_{2}^{0}f_{2}^{S}X_{2}^{S}$$

The coefficients of activity at the interface are $f_{1}^{S}$ and $f_{2}^{S}$ and the $C_{1}^{0}$ and $C_{2}^{0}$ are the molar concentrations at which pure amphiphiles achieve a surface tension for single amphiphiles. It is possible to approximate the activity coefficients at the interface of non-ideal mixed systems by the following expression:

(17) $$lnf_{1}^{S}=\beta^{s}{\left(X_{2}^{S}\right)}^{2}$$

(18) $$lnf_{2}^{S}=\beta^{s}{\left(X_{1}^{S}\right)}^{2}$$

The values of $X_{1}^{S}$ is calculated by solving Equation (19) iteratively:

(19) $$\frac{{\left(X_{1}^{s}\right)}^{2}\ln\left(\alpha_{1}C_{mix}/X_{1}^{s}C_{1}\right)}{{\left(1-X_{1}^{S}\right)}^{2}\ln\left[\left(1-\alpha_{1}\right)C_{mix}/\left(1-X_{1}^{S}\right)C_{2}\right]}=1$$

where the mutual interaction between two amphiphiles at the interface is designated by a parameter, $\beta^{s}$ as:

(20) $$\beta^{s}=\frac{\ln\left(\alpha_{1}C_{mix}/X_{1}^{S}C_{1}\right)}{{\left(1-X_{1}^{S}\right)}^{2}}$$

The values of $X_{1}^{S}$, $\beta^{s}$, $f_{1}^{S}$ and $f_{2}^{S}$ are listed in Table 4. The $\beta^{s}$ values for current mixed systems are negative confirm synergistic interaction at the interface.

In the consideration of morphology of a micelle, Israelachvili [53] showed that the geometry of the micellar aggregate can be estimated by the value of the packing parameter (*P*) as:

(21) $$P=v/Al_{c}$$

where *v* is the volume of the hydrophobic chain, *l*_c_ is the maximum effective length and *A* is the surface area of the polar head group. The *A* values are difficult to calculate so in the calculation we used *A_min_* values instead of *A*. The values of *v* and *l*_c_ can be computed by the Tanford formula [54]:

(22) $$v=27.4+2.69N_{c}$$

(23) $$l_{c}=1.54+1.265N_{c}$$

The values of the packing parameter (*P*) are listed in Table 3. The *P* values determine micelle shape or geometry. For spherical, nonspherical, vesicles, and inverted the values are <1/3, <1/2, <1, and >1, respectively. For our system spherical micelle are formed by the TCH + 2G4 mixed systems. However, for TCH + TTAB mixed systems spherical and nonspherical geometry is observed.

#### 2.2.2. Thermodynamics Parameters at Interface

The standard free energy of interfacial adsorption at the interface has been evaluated from the relation [55]:

(24) $$\Delta G_{add}^{o}=\Delta G_{m}^{o}-\left(\frac{\pi_{CMC}}{\Gamma_{max}}\right)$$

The surface pressure at the cmc here designated by the term $\pi_{CMC}$. The parameter $G_{m}^{o}$ used in Equation (24) is the standard Gibbs free energy and calculated by the following Equation (25) [56]:

(25) $$\Delta G_{m}^{o}=RTlnX_{CMC}$$

It was found that the achieved $\Delta G_{add}^{o}$ values of are –ve (Table 4) such as those of $\Delta G_{m}^{o}$, however, the magnitude of $\Delta G_{add}^{o}$ is much greater, indicating that the adsorption is more spontaneous for current mixed systems.

Activity coefficients values can be used to calculate $G_{exc}^{s}$ (excess free energy of micellization) at interface:

(26) $$G_{exc}^{s}=RT\left[X_{1}^{S}lnf_{1}^{S}+\left(1-X_{1}^{S}\right)lnf_{2}^{S}\right]$$

Following the negative values of $G_{exc}^{s}$ (Table 4), stability may be achieved by the steady mixing at the interface, that was not acquired by the monolayer of single amphiphiles. The –ve values of $G_{exc}^{s}$ also indicate synergistic interaction at the interface. An energetic parameter, $G_{min}^{s}$, is also able to quantify the extent of synergism following the mixing of one or more amphiphiles [57],

(27) $$G_{min}^{s}=A_{min}\gamma_{CMC}N_{A}$$

The work involved in creating a surface is per mole of a solution and the transition from bulk phase to the interface can be defined by the above energetic parameter. Based on the low values of $G_{min}^{s}$ in Table 4, it appears that a more energetically steady surface is created, and extra surface activity is achieved.

## 3. UV-Visible Study of the Mixed System

The binding and complex formation between two components can be monitored by electronic absorption. Two absorption peaks (at 226 and 310 nm) were seen on the UV-visible spectra of a drug (TCH). The first peak at 226 nm may be due to the π–π* and the second one (310 nm) due to the n–π* transitions [32,58]. It is worth explaining here that the surfactants did not show any peak in the UV-visible spectrum. The presence of the aminobenzoate group in the drug molecules (Figure 1) is responsible for the characteristic peak. The UV-visible spectrum of TCH with increasing concentration of surfactants is shown in Figure 2. The intensity of UV-visible spectra in the presence of surfactants shows a minor increase in intensity while wavelength remains unchanged. The 2G4 + TCH system shows a greater increase in intensity than TTAB + TCH. This could be due to the positive charge on the head groups of surfactants and drug repelling drug molecules. Therefore, complexation was not observed between drug and surfactant molecules. Sharma and Mahajan the same behavior of trifluoperazine dihydrochloride(TFP) with cationic surfactant has been observed [6].

## 4. Conclusions

The current work deals with the mixed micellization study of tetracaine hydrochloride (anesthetic amphiphilic drug) with the gemini surfactant and its monomeric surfactant in the aqueous medium. Surface tension and UV-visible spectroscopy techniques were used in this study. The following conclusions can be drawn from the results.

1. The cmc values of pure amphiphiles were matched with the literature values. The lower experimental values of cmcs than the ideal one confirm non-ideal behavior.
2. The negative values of interaction parameter ($\beta^{Rub}$), indicates a favorable environment for mixed micelle formation. A higher level of attractive interaction (synergism) was observed with the 2G4 + TCH mixed system due to the higher hydrophobicity of gemini molecules.
3. For the TTAB + TCH mixed system the values of $A_{min}$ are lower than the drug, this shows that more tightly packed amphiphiles curvature is formed. For the 2G4 + TCH mixed system the values of $A_{min}$ are higher than the TCH, indicate loosely packed monomer interface is formed. When the chain lengths of amphiphiles are different, the one component above the height of the adjacent component will create thermal motion. Due to this thermal motion, the interface is disturbed and as a result, loose packing is created ($\Gamma_{max}$ decreases and $A_{min}$ increases).
4. As $\Delta G_{m}^{o}$ and $\Delta G_{add}^{o}$ were negative values, specifies micelles formation and surfactant adsorption at air-water interface are energetically advantageous, while a –ve values of $G_{exc}^{s}$ indicates the energetic stabilization accompanied by mixed monolayer formation.
5. For current binary mixed systems, the positive values of entropy confirmed the entropy contribution must be the driving force of mixed micellization.

## 5. Experimental

### 5.1. Materials and Methods

**Scheme 2:**
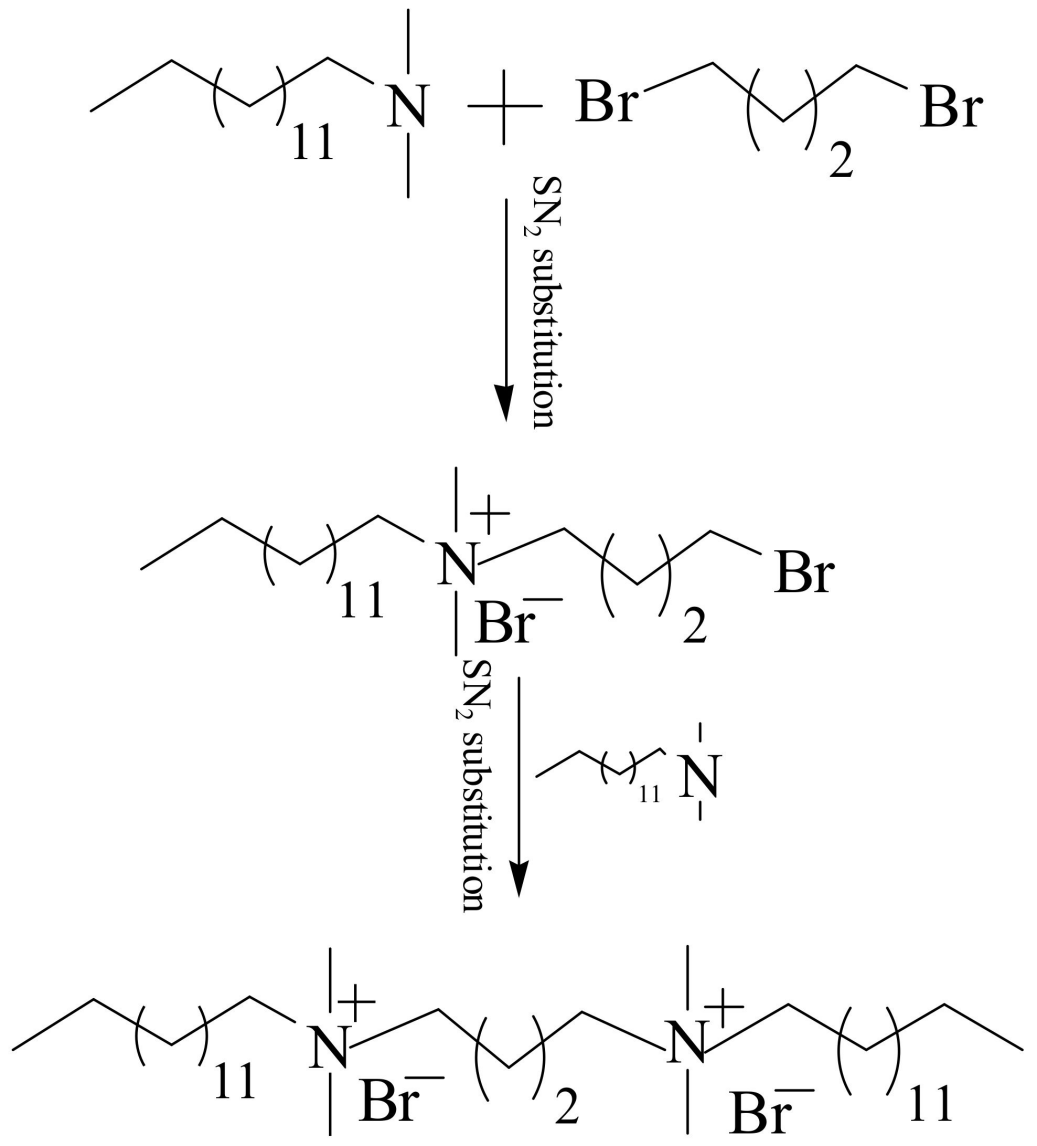
Protocol for gemini surfactant (*N*,*N*′-bis(dimethyl tetradecyl)-α,ω-alkane diammonium bromide, 2G4) synthesis.

The anesthetic amphiphilic drug, tetracaine hydrochloride (TCH) was the product of Molecules On (Basel, Switzerland) and used as received. Tetradecyl trimethyl ammonium bromide (TTAB) was procured from Merck (Kenilworth, NJ, USA). N,N-dimethyl tetradecyl amine (Sigma-Aldrich, St. Louis, MO, USA), 1,4-dibrobutane (Sigma-Aldrich), absolute ethanol (Sigma-Aldrich), ethyl acetate (Sigma-Aldrich) were purchased for gemini synthesis. The synthesis of gemini surfactant was followed by the earlier procedure [33,34] and protocols are presented in Scheme 2.

### 5.2. Surface Tension Measurements

The tensiometric titrations were carried out at fixed temperature (298.15 K) by using Attention tensiometer (Sigma 701, Darmstadt, Germany), and obeying the duNouy ring detachment method. The ring was overheated on an ethanol flame and wash with distilled water before each measurement. Each experiment was repeated at least three times to safeguard reliable output. A thermostat was connected to flow the water and maintain the desired temperature equilibration. To weigh the compounds for solution preparation Citizen CX-220 analytical balance was used. De-ionized ultra-pure water was used in the making of all solutions.

### 5.3. UV-Visible Spectroscopy Measurements

The UV-visible spectra of TCH in the absence and presence of surfactants were recorded by using Thermo Scientific Spectrophotometer (Evolution 300, Waltham, MA, USA). All data were obtained in the range of 200 to 400 nm (Figure 2). The stock solution of the drug was prepared in ultra-pure water while the stock solution of surfactants was made using previously made TCH solution to observe the effects of surfactants on UV-visible spectra of the drug. To obtain better results the blank was subtracted from the drug spectra before analysis.

## Figures and Tables

**Table 1:** Mixed micellar parameters of TCH + TTAB/2G4 mixtures at *T* = 298.15 K.

α_1_	*cmc* (mM)	cmc* (mM)	$X_{1}^{Rub}$	$X_{1}^{\mathrm{ideal}}$	−*β^Rub^*	$f_{1}^{Rub}$	$f_{2}^{Rub}$
TCH + TTAB							
0.0	93.00						
0.1	10.20	23.37	0.606	0.774	3.79	0.555	0.249
0.3	8.81	9.36	0.884	0.929	0.72	0.990	0.570
0.5	4.69	5.85	0.835	0.968	2.70	0.929	0.152
0.7	3.49	4.26	0.862	0.986	3.38	0.938	0.081
0.9	3.18	3.34	0.957	0.996	4.61	0.991	0.015
1.0	3.02						
TCH + 2G4							
0.0	93.00						
0.1	0.57	1.46	0.716	0.986	7.78	0.534	0.018
0.3	0.33	0.49	0.825	0.996	6.26	0.825	0.014
0.5	0.24	0.29	0.876	0.998	6.30	0.906	0.007
0.7	0.16	0.21	0.874	0.999	7.17	0.893	0.004
0.9	0.13	0.16	0.885	0.999	8.63	0.893	0.001
1.0	0.15						

α1 = TTAB or 2G4 molar ratio in the binary amphiphile mixture, *cmc* = experimental critical micelle concentration, cmc* = ideal critical micelle concentration, $X_{1}^{Rub}$ = mole fraction of component 1 in the mixed micelle, $X_{1}^{\mathrm{ideal}}$ = ideal mole fraction of component 1 in the mixed micelle, −*β^Rub^* = interaction parameter, $f_{1}^{Rub}$ = activity coefficient of component 1, $f_{2}^{Rub}$ = activity coefficient of component 2.

**Table 2:** Energetic constraints of TCH + TTAB/2G4 mixtures at *T* = 298.15 K.

α_1_	$-G_{mix}^{E}/-\Delta H_{M}$ (kj·mol^−1^)	−Δ*G_M_* (kj·mol^−1^)	−Δ*G_M_*^Ideal^ (kj·mol^−1^)	*T*Δ*S_M_* (kj·mol^−1^)	$\left|\frac{T\Delta S_{M}}{\Delta G_{M}}\right|$
TCH + TTAB					
0.1	2.24	3.97	1.66	5.80	1.46
0.3	0.18	1.09	0.89	3.05	2.79
0.5	0.92	2.06	1.11	3.84	1.86
0.7	0.99	2.02	0.99	3.44	1.71
0.9	0.47	0.92	0.44	1.53	1.65
TCH + 2G4					
0.1	3.92	5.49	1.47	5.27	0.96
0.3	2.24	3.45	1.15	4.05	1.17
0.5	1.72	2.69	0.92	3.26	1.21
0.7	1.95	2.94	0.94	3.31	1.13
0.9	2.17	3.10	0.88	3.13	1.01

α_1_ = TTAB or 2G4 molar ratio in the binary amphiphile mixture, *G^ex^*/Δ*H_M_* = excess free energy of mixed micellization/enthalpy change for binary mixed system, Δ*G_M_* = Gibbs free energy change for micellization, Δ*G_M_*^Ideal^ = Gibbs free energy change for ideal binary mixed system, Δ*S_M_* = entropy change for a binary mixed system.

**Table 3:** Surface and packing parameters of TCH + TTAB/2G4 mixtures at *T* = 298.15 K.

α_1_	10^6^ Γ_*max*_ (mol·m^−2^)	*A*_*min*_ (Å^2^)	*pC*_20_	γ_*cmc*_ (mN·m^−1^)	π_*cmc*_ (mN·m^−1^)	*P*
TCH + TTAB						
0.0	2.49	66.57	39.35	40.42	31.58	0.31
0.1	5.66	29.31	6.37	37.38	34.62	0.72
0.3	5.39	30.80	5.14	36.43	35.56	0.68
0.5	5.84	28.41	2.76	35.25	36.75	0.74
0.7	5.26	31.58	1.67	33.96	38.04	0.67
0.9	2.97	55.94	0.89	32.73	39.27	0.38
1.0	1.97	83.98	0.34	28.43	43.57	0.25
TCH + 2G4						
0.0	2.49	66.57	39.35	40.42	31.58	0.31
0.1	2.11	78.60	0.27	41.14	30.855	0.27
0.3	1.91	87.02	0.13	41.36	30.64	0.24
0.5	1.69	98.03	0.08	40.81	31.19	0.21
0.7	1.73	95.84	0.06	41.45	30.55	0.22
0.9	2.02	82.07	0.05	40.79	31.21	0.26
1.0	1.77	93.62	0.06	39.08	14.68	0.23

α_1_ = TTAB or 2G4 molar ratio in the binary amphiphile mixture, Γ_max_ = surface excess, *A*_min_ = minimum area per molecule, γ*_cmc_* = molar concentration of amphiphiles at *cmc*, π_cmc_ = surface pressure at cmc, *P =* packing parameter.

**Table 4:** Mixed interfacial and thermodynamic parameters TCH + TTAB/2G4 mixtures at interface.

α_1_	$X_{1}^{s}$	$-\beta^{s}$	$f_{1}^{s}$	$f_{2}^{s}$	$-G_{ex}^{s}$ (kj·mol^−1^)	$-\Delta G_{m}^{o}$ (kj·mol^−1^)	$-\Delta G_{add}^{o}$ (kj·mol^−1^)	G*_min_* (kj·mol^−1^)
TCH + TTAB								
0.0						15.83	28.50	16.21
0.1	0.71	2.56	0.801	0.278	1.32	21.32	27.43	6.59
0.3	0.88	1.76	0.973	0.258	0.47	21.67	28.27	6.76
0.5	0.91	2.17	0.983	0.164	0.43	23.24	29.53	6.03
0.7	0.92	2.90	0.984	0.083	0.50	23.96	31.20	6.46
0.9	0.94	4.01	0.986	0.028	0.54	24.19	37.43	11.03
1.0						24.33	46.37	14.38
TCH + 2G4								
0.0						15.83	28.50	16.21
0.1	0.74	6.92	0.618	0.023	3.33	28.47	43.08	19.47
0.3	0.81	6.76	0.784	0.012	2.57	29.86	45.92	21.67
0.5	0.86	6.58	0.875	0.008	1.99	30.62	49.04	24.09
0.7	0.87	7.48	0.874	0.004	2.15	31.53	49.17	23.92
0.9	0.91	7.64	0.945	0.002	1.48	32.12	47.55	20.16
1.0						31.80	50.36	22.03

α_1_ = TTAB or 2G4 molar ratio in the binary amphiphile mixture, $X_{1}^{s}$ = micellar mole fraction of component 1 at interface, $\beta^{s}$ = interaction parameter at the interfacial monolayer, $f_{1}^{s}$ = activity coefficients at the surface, $f_{2}^{s}$ = activity coefficients at the surface, $G_{ex}^{s}$ = excess free energy, $\Delta G_{m}^{o}$= standard Gibbs free energy, Δ*G_ads_* = standard free energy of interfacial adsorption, *G_min_* = free energy at the surface.

## Data Availability

Not applicable.
